# Double restriction-enzyme digestion improves the coverage and accuracy of genome-wide CpG methylation profiling by reduced representation bisulfite sequencing

**DOI:** 10.1186/1471-2164-14-11

**Published:** 2013-01-16

**Authors:** Junwen Wang, Yudong Xia, Lili Li, Desheng Gong, Yu Yao, Huijuan Luo, Hanlin Lu, Na Yi, Honglong Wu, Xiuqing Zhang, Qian Tao, Fei Gao

**Affiliations:** 1 Science & Technology Department, BGI-Shenzhen, No.11, Bei Shan Industrial Zone, Yantian District, Shenzhen, China; 2 Cancer Epigenetics Laboratory, Department of Clinical Oncology, State Key Laboratory of Oncology in South China, Sir YK Pao Center for Cancer and Li Ka Shing Institute of Health Sciences, The Chinese University of Hong Kong, Shatin, NT, Hong Kong, SAR, China

**Keywords:** Single-enzyme RRBS, Double-enzyme RRBS, DNA methylation, CpG coverage

## Abstract

**Background:**

Reduced representation bisulfite sequencing (RRBS) was developed to measure DNA methylation of high-CG regions at single base-pair resolution, and has been widely used because of its minimal DNA requirements and cost efficacy; however, the CpG coverage of genomic regions is restricted and important regions with low-CG will be ignored in DNA methylation profiling. This method could be improved to generate a more comprehensive representation.

**Results:**

Based on *in silico* simulation of enzyme digestion of human and mouse genomes, we have optimized the current single-enzyme RRBS by applying double enzyme digestion in the library construction to interrogate more representative regions. CpG coverage of genomic regions was considerably increased in both high-CG and low-CG regions using the double-enzyme RRBS method, leading to more accurate detection of their average methylation levels and identification of differential methylation regions between samples. We also applied this double-enzyme RRBS method to comprehensively analyze the CpG methylation profiles of two colorectal cancer cell lines.

**Conclusion:**

The double-enzyme RRBS increases the CpG coverage of genomic regions considerably over the previous single-enzyme RRBS method, leading to more accurate detection of their average methylation levels. It will facilitate genome-wide DNA methylation studies in multiple and complex clinical samples.

## Background

Through a process of post-replicative covalent modification, 5-methylcytosine is considered as a “fifth base” in mammals. 5-methylcytosine is formed by the addition of a methyl group to the 5-position of a cytosine ring and is catalyzed by DNA methyltransferases (DNMTs) [1]. The correct localization of this modification on the chromosomes is crucial for diverse biological processes, such as embryonic development, genomic imprinting and X-chromosome inactivation [2-4]. Aberrant DNA methylation is associated with many human diseases, including cancers [5].

Multiple methods have been developed to probe the distribution of 5-methylcytosine on a genome-wide scale. The Infinium Methylation 450 K array [6,7], based on quantitative genotyping of the C/T polymorphism generated by bisulfite conversion, is now widely used because of its cost-efficiency. However, its CpG coverage is limited. Next generation sequencing-based methods can detect extensive genome-wide cytosine methylation. These methods can be classified into two categories. Firstly, bisulfite-treatment-based approaches, including whole genome bisulfite sequencing (WGBS) [8,9], reduced representation bisulfite sequencing (RRBS) [10], and target-region capture followed by a bisulfite sequencing strategy, which we and others developed recently [11,12]. Secondly, affinity-purification-based approaches, including MeDIP-Seq [13] and MBD-Seq [14,15]. Among the above-mentioned methods, WGBS has been widely used to profile DNA methylation at single-base resolution genome wide; however it consumes micrograms of genomic DNA at a high cost, which is not feasible for many clinical samples, such as tumors obtained by laser capture microdissection or rare stem cell populations [16]. RRBS representatively interrogates DNA methylation at single-nucleotide resolution in genomic regions that are especially enriched for CpG islands (CGIs) and promoters. Promoter-associated CGIs represent important *cis* regulatory elements in the human genome: ~70% of annotated gene promoters are associated with a CGI [17] and about half of all CGIs contain transcription start sites (TSSs) [18,19]. Studies of cancer genomes also reveal that aberrant methylation of promoter-associated CGIs is acquired during tumorigenesis [5,20]. Promoter-associated CGIs are therefore of special interest to biomedical researchers. RRBS is the first choice for DNA methylation analysis of clinical samples because of its minimal DNA requirements and low cost, based on reduced representation of the genome.

However, the majority of intergenic regions and CGI shores are beyond the detection range of current RRBS strategies [16,21], and DNA methylation in these regions also plays important roles in various biological processes. Methylation of intergenic or intragenic regions has been suggested to be involved in regulating alternative splicing [22,23] and expression of non-coding RNAs (ncRNA), such as miRNAs and snoRNAs in tumorigenesis [20,24,25]. Methylation of CGI shores might also be important as tissue-specific differential methylation regions (T-DMRs) and cancer-specific differential methylation regions (C-DMRs), both of which are preferentially located in CGI shores [22,26]. Furthermore, although RRBS can representatively interrogate nearly 65% of all promoters, it can only cover about 30% of the CpG dinucleotides of the promoters detected, which may not sufficiently represent their actual methylation levels [16,21].

Based on *in silico* simulation of enzyme digestion on human and mouse genomes, we improved the current single-enzyme (*MspI*) RRBS (sRRBS) strategy by adding another enzyme (*ApeKI*) to interrogate more representative regions. We applied this double-enzyme strategy (dRRBS) to a lymphoblastoid (YH) cell line and a mature dendritic (mDC) cell line [11], and confirmed that the CpG coverage of CGIs, CGI shores, promoters and introns were considerably increased. Furthermore, the average methylation levels in genomic regions varied along with increasing CpG coverage, indicating that the dRRBS strategy can more accurately reflect their average methylation levels. Additionally, we comprehensively characterized the DNA methylation profiles of a colorectal carcinoma cell line pair (HCT116 and DKO, which was generated through double knock-out of *DNMT1* and *DNMT3b* in HCT116) [27]. As expected, genome-wide demethylation in DKO cells was observed. Surprisingly, we also observed that DNA methylation of certain regions was maintained, suggesting a selection mechanism for cancer cells’ survival, as reported previously [28]. In summary, the improved dRRBS strategy will increase CpG coverage of genomic regions and improve accuracy in detecting their average methylation levels, thus aiding future methylome studies of diverse clinical samples.

## Results

### Design of the double-enzyme RRBS method

Recently, two groups systematically assessed the sRRBS technology and demonstrated that it is able to enrich the promoter and CGI regions [29,30]. We further applied pair-end sequencing with a 50 bp read length (PE50) strategy to increase the cytosine coverage, but the proportion of detected CpG dinucleotides within other genetic elements (e.g. functional regions, such as, the promoter, 5-UTR, and CDS) were still low. sRRBS has several advantages (e.g. in detection of formalin-fixed, paraffin-embedded samples or minimal amount of starting genomic DNA) [16]; therefore, we sought to improve the technology by adding another restriction enzyme to further fragment the genome. By proper size-selection, the number of CpGs detected and the coverage of other genomic regions, such as CGI shores and introns can be further increased. We performed *in silico* simulation of enzyme digestion on the human genome (hg18) and the mouse genome (mm9) by *MspI* combined with other methylation insensitive restriction endonucleases, including *HpyCH4V*, *AluI*, *BstNI*, *HaeIII*, *HpyCH4III*, *ApeKI*, *BanII*, *BglII*, *TaqαI*, *SphI*, *BamHI*, *BssSI* and *KpnI*. Two different ranges of size-selection (40-220 bp or 40-300 bp) for *in silico* digested DNA fragments were evaluated. The interrogated CpG dinucleotides, based on 50 bp forward reads, were generally increased on different genomic elements by double-enzyme digestion in comparison with single-enzyme *MspI* digestion in both human and mouse genomes (Additional file 1: Table S1).

Depending on the specific experimental requirements, researchers can select an appropriate combination of enzymes for the RRBS library construction. The recognition sites of *MspI* are mostly located in high-CG regions [16]; therefore, the *MspI*-digested DNA will be further fragmented by adding an enzyme with CG inside its recognition sites; however, the genomic coverage on regions with low CG density was only slightly increased (exemplified by *TaqαI* (T|CGA) and *BssSI* (C|ACGAG), Additional file 1: Table S1). A more representative coverage in low-CG regions can be achieved using an enzyme such as *ApeKI*, which has no CG inside its recognition site. It is clear the cost rises inline with improvement in CpG coverage; thus, considering both increased coverage and cost, we chose a combination of *MspI* and *ApeKI* digestion to test the feasibility of the dRRBS strategy. This strategy achieves an approximately two-fold increase in CpG coverage. Other combinations, such *MspI* and *BstNI*, also increase CpG coverage, but with more sequencing required (Additional file 1: Table S1). Moreover, the PE90 (with read length of 90 bp) sequencing strategy can cover more CpG sites than the PE50 sequencing strategy, based on current high-throughput platforms, such as Illumina Hiseq2000. However, the PE50 sequencing strategy is more cost-effective, considering the amount of data required (Additional file 1: Table S2). Thus, in present study, we applied a PE90 sequencing strategy to generate data, which can be further excised into PE50 sequencing data by cutting off 50 bp from the sequencing reads *in silico*. We then systematically evaluated the CpG coverage and data requirements from different sequencing strategies of the dRRBS approach.

### Data generation and coverage evaluation

Using a double-enzyme (*MspI* plus *ApeKI*) RRBS strategy for genome-wide DNA methylation detection, we constructed sequencing libraries from the genomic DNA of YH (with 40-220 bp insert fragments) and mDC cell lines (with 40-300 bp insert fragments). We then compared the sequencing results to sRRBS results with 40-220 bp fragments digested by *MspI* (newly generated for the mDC cell line, but previously generated for YH [21]). As a result, 64.84 M (YH) and 71.73 M (mDC) uniquely aligned high-quality PE90 reads with an average of 10× sequencing depth were generated by dRRBS, while 63.45 M (YH) and 98.71 M (mDC) uniquely aligned PE50 reads with an average of 20× sequencing depth were generated by sRRBS [21] (Additional file 1: Table S3).

**Figure 1:**
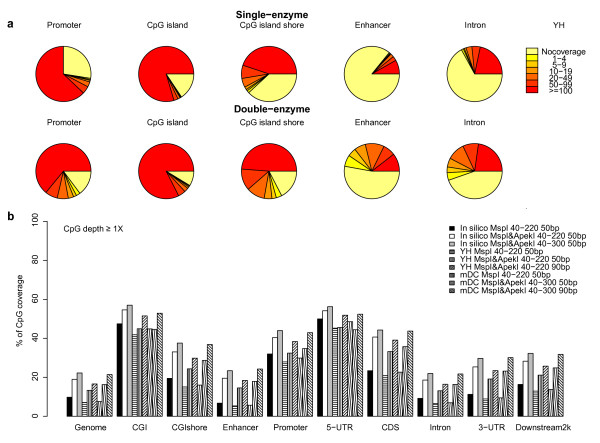
**CpG coverage in different genomic regions detected by the single-enzyme (*MspI*) and double-enzyme RRBS (*MspI *plus *ApeKI*) methods.** (**a**) Genomic coverage by single-enzyme (top) and double-enzyme (bottom) RRBS in the YH genome with the same size selection (40-220 bp) and the same read lengths (50 bp). The number of individual CpG dinucleotides measurements in each genomic element, including promoters (2 kb upstream to TSS), CGIs and CGI shores (2 kb adjacent to upstream and downstream of CGIs) are displayed by incremental colors. (**b**) Genomic CpG coverage (CpG was covered at least once, CpG depth ≥ 1×) by single- or double-enzyme RRBS based on *in silico* analysis or sequencing data in YH and mDC samples. Ranges of size-selections for enzyme digested fragments and read lengths are displayed.

To evaluate the increased coverage of CpGs in specific genomic regions caused by addition of *ApeKI* digestion, we excised the PE90 reads for YH generated by dRRBS into 50 bp reads, and compared the data with sRRBS. Two types of measurements were applied in parallel. One approach simply counted the numbers of individual CpGs, as suggested in the original RRBS method [16]. We examined different genomic regions of the YH sample, including promoters, CpG islands, CGI shores, enhancers and introns (Figure 1a), as well as four other types of genomic region (5-UTR, CDS, 3-UTR and regions downstream of genes) (Additional file 2: Figure S1). A considerable increase in coverage for all these regions, especially 6.6% for promoters, 5.7% for CpG islands, 13.0% for CGI shores and 10.6% for introns were detected, with more than 25 individual CpGs measurements (corresponding to the sum of high-quality sequencing coverage over all CpG dinucleotides in a region), as described previously [16]. The second method was to calculate and compare the percentage of CpG dinucleotides detected. As indicated in Figure 1b, the coverage of total CpGs was nearly doubled (13.32% in dRRBS in comparison with 7.08% in sRRBS) in the YH genome by PE50 sequencing with 40-220 bp size selection. Accordingly, the CpG coverage within different genomic elements largely increased, especially for CGI shores and gene bodies. Furthermore, applying a sequencing strategy with longer reads or choosing a wider range of size-selection of digested fragments would expand the coverage of CpG sites, as exemplified in the comparison between different strategies using the mDC sample (Figure 1b). Such scale of increased coverage might enable more accurate analysis of DMRs, not only for large regions, as exemplified by one randomly selected region across chr7:98,550,000-99,050,000 (Additional file 2: Figure S2a), but also for specific genes, as exemplified by two tumor suppressor genes (TSGs) with CGI and one randomly selected gene without CGI. In particular, we clearly show that dRRBS detected more CpG of CGIs in promoters of the *TBX6* and *IRF8* genes than sRRBS (Additional file 2: Figure S2b).

As the genome-wide coverage of CpG sites increased, one major concern would be whether the sequencing cost would also increase. Therefore, we estimated the cost efficiency in term of reads per informative CpG measurement. For CpGs with more than 1× and 5× sequencing depth, reads per informative CpG were 0.99 and 1.06, respectively, for dRRBS based on the same size selection (40-220 bp) and read length (PE50) in the YH sample. For sRRBS, values of 0.83 and 0.85 were observed, indicating that the cost efficiency is similar between the two methods. Indeed, the average sequencing depth of dRRBS would be less than that for sRRBS given similar amount of raw data (Additional file 1: Table S3). However, taking CpG sites with 5× and 10× sequencing depth from both dRRBS and sRRBS, we observed that 3.73% and 2.24% of additional CpGs could be detected by dRRBS, respectively, with the same size selection (40-220 bp) and read length (PE50) in the YH sample (Additional file 2: Figure S3a, S3b). These results suggest that dRRBS is more applicable in genome-wide studies of DNA methylation with more representative CpG coverage, especially considering recent rapidly decreasing costs of high-throughput sequencing.

### Accuracy and efficiency of methylation detection of DMRs

To evaluate the accuracy of dRRBS, we used whole genome bisulfite sequencing (WGBS) data for the YH [31] and mDC [11] samples that were previously generated. We used data with at least five-fold sequencing depth to assess the methylation status of individual CpG dinucleotides by Pearson correlation analysis. A general consistency of methylation levels of CpGs was observed between RRBS and WGBS, in which the Pearson correlation coefficients were similar for the two RRBS methods (Additional file 2: Figure S4, Additional file 1: Table S4).

**Figure 2:**
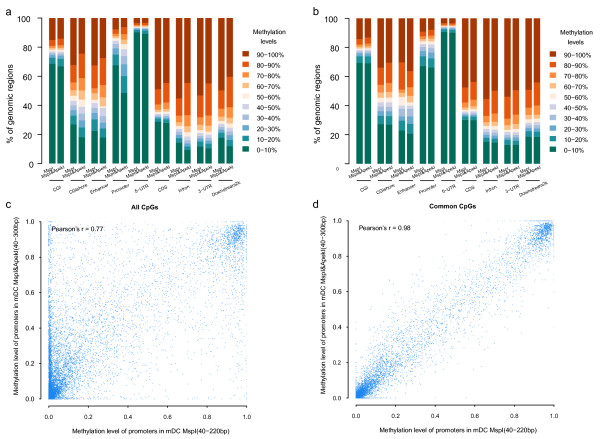
**Comparison of methylation levels for different genomic regions detected by single- or double-enzyme RRBS in mDC samples.** Distribution and comparison of methylation levels in different genomic regions with all CpGs (**a**) or with commonly detected CpGs (**b**), detected by the two strategies. A scatter plot depicting methylation levels of promoters with all CpGs (**c**) or with commonly covered CpGs (**d**) detected by the single- or double-enzyme RRBS strategies. Each dot represents one promoter region.

As more CpGs within each genomic element were detected by the dRRBS method and different CpG coverage in a genomic element generated different distributions of CpG methylation when comparing sRRBS with dRRBS (Additional file 2: Figure S5), we were interested in whether there was a discrepancy in the detection of general methylation levels of a genomic element by the two strategies. We described the average methylation levels of elements using sRRBS and dRRBS data sets of the mDC sample. Interestingly, the methylation levels of elements extracted by the two strategies showed greater differences in the promoter, CGI shore and intron regions than for common CpGs (CpGs that covered by both sRRBS and dRRBS) in those regions (Figure 2a, 2b). The methylation accuracy of CpG sites was confirmed by the Pearson correlation coefficients (Additional file 1: Table S4). Consistent results were also observed in scatter analysis (Figure 2c, 2d, Additional file 2: Figure S6). Taking promoters as an example, the Pearson correlation coefficient of methylation between the two strategies was 0.77 (Figure 2c), while the correlation coefficient was increased to 0.98 when the common CpGs were analyzed (Figure 2d).

As averaging over more CpGs is likely to be more accurate, we then selected the genomic features with more CpG sites enriched by dRRBS than sRRBS, and compared their methylation levels with WGBS. Generally, higher Pearson correlation coefficients between dRRBS and WGBS were observed than the values for sRRBS, indicating that dRRBS is more accurate (Additional file 2: Figure S7). These results indicated that the average methylation levels could be biased by the coverage of CpGs in specific genomic regions when detected by techniques with different scales of representation. It is important to achieve a full coverage of CpGs to get an accurate estimation of regional DNA methylation.

### Genome-wide profiling of DNA methylation in HCT116 and DKO cell lines

**Figure 3:**
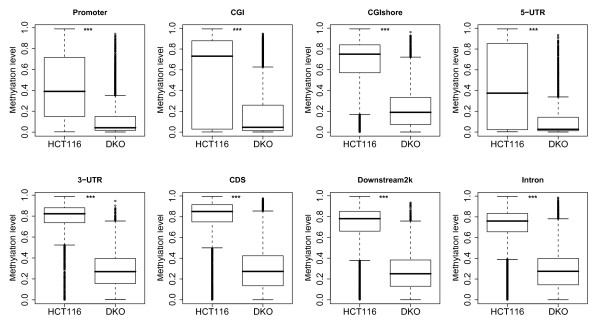
**DNA methylation levels of different genomic elements in colorectal carcinoma cell lines HCT116 and DKO.** Box plots based on the distribution of DNA methylation levels measured by double-enzyme RRBS are shown; differential methylation was tested by the *chi*-square test (***, *P*<0.001).

Colorectal carcinoma cell line HCT116 and its derivative cell line DKO, which bears homozygous deletions of both *DNMT3b* and *DNMT1*, are widely used models to study target genes of DNA methylation-mediated silencing, especially for tumor suppressor genes (TSGs) [32-36] and miRNAs [24,37]. Researchers also observed that a few key regions preferentially maintain their methylation when global DNA methylation is artificially reduced in DKO cells, indicating that cancer cells might depend on DNA methylation for survival [28]. However, genome-wide methylation status accompanied with gene expression profiling has not yet been completed. The full methylome profile for these two cell lines would provide basic data for researchers to fully address the above-mentioned issues. We then used the dRRBS method to analyze the methylation status coupled with gene expression profiling by digital gene expression (DGE) for the paired cell lines (Additional file 1: Table S5). As expected, large amounts of hypomethylation in different genomic elements were present in DKO cells compared with HCT116 cells (Figure 3). A total of 4542 out of 11391 genes showed significantly different levels of DNA methylation within their promoter regions (*P*-value < 0.01, methylation level differences between two cell lines was greater than 20%). Among these genes, 4526 genes were significantly hypomethylated in DKO cells compared with HCT116 cells. Furthermore, 372 of the hypomethylated genes were significantly upregulated in DKO cells (Additional file 1: Table S5). Functional analyses revealed that these 372 genes were enriched in a broad spectrum of KEGG pathways, including those related to cancer in the MAPK signaling pathway and Jak-STAT signaling pathway (Additional file 1: Table S6). This result suggested that some of these 372 genes might be tumor suppressor genes (TSGs). Thus, the global DNA methylation, as well as local methylation, in most of the methylated genes in the parental HCT116 cells were decreased in DKO cells, as a consequence of the impaired DNA methyltransferase machinery. Despite of that, some regions maintained high methylation levels in DKO cells (Figure 3). In particular, promoter regions of 16 genes remained significantly hypermethylated in DKO compared with HCT116 cells (Additional file 1: Table S5). Previously, it was reported that DKO cells are under constant selective pressure to retain DNA methylation at some key regions to survive [38]. These hypermethylated genes in DKO cells detected by the dRRBS method might be crucial for cancer cells’ survival.

### Validation of promoter methylation by bisulfite-PCR sequencing

Previously, we identified a series of functional tumor suppressors that are frequently methylated in multiple tumors. Using the improved RRBS method, we tested certain known TSGs (Additional file 1: Table S7), including *WNT5A*[32], *PCDH10*[33], *DLC1*[34], *IRF8*[35] and *ZNF382*[36]. The results of the expanded RRBS method confirmed that all these genes are relatively hypermethylated in HCT116, but hypomethylated in the DKO cell line (Additional file 1: Table S7). Hence the dRRBS provides a robust platform for examining DNA methylation changes and will be useful in future applications for identifying TSGs with aberrant methylation.

**Figure 4:**
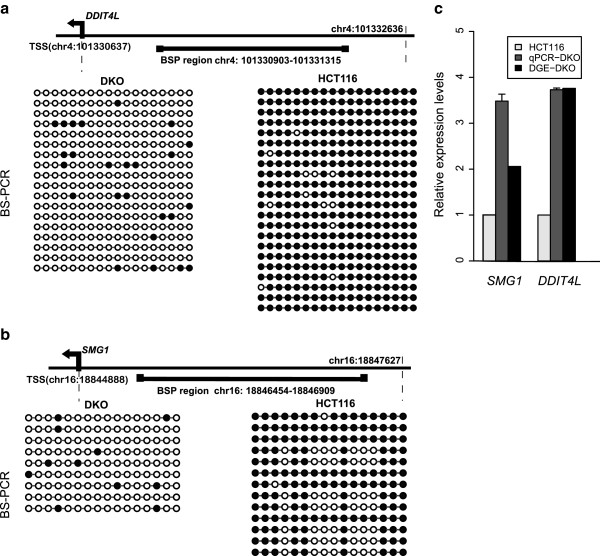
**Validation of methylation status of selected regions in promoters and the expression of their associated genes.** (**a**-**b**) Methylation statuses of specific regions of two cell lines identified by Bisulfite genomic sequencing. Each circle represents one CpG site within the tested sequence: filled circles represent methylated CpGs while open circles represent unmethylated CpG sites. (**a**) Region located in the promoter region of *DDIT4L* gene. (**b**) Region located in the promoter region of *SMG1* gene. (**c**) Gene expression levels in DKO relative to HCT116 cell line. The results are presented as real time PCR data, normalized by β-actin expression levels and digital gene expression sequencing (DGE) methods. Error bars denote S.D.

Using bisulfite genomic sequencing with multiple clones, we selected two regions in promoters for further validation. We confirmed that both selected regions were hypermethylated in HCT116, but hypomethylated in DKO, consistent with the dRRBS results (Figure 4a, 4b, Additional file 1: Table S8). Concomitantly, the relative expression levels of the differential methylation regions (DMR)-associated genes were validated by RT-qPCR and normalized by β-actin expression. Both RT-qPCR and DGE results suggested that the transcription of these genes was activated when promoter demethylation was achieved through knockout of both *DNMT1* and *DNMT3b* in DKO cells (Figure 4c). *DDIT4L* was previously studied in melanoma patients as a risk factor, with a hypermethylated promoter [39]. In the present study, we found that the expression of *DDIT4L* was low and it had a hypermethylated promoter in HCT116, but was reactivated in DKO cells, where it showed a decreased methylation level, suggesting an aberrant methylation status of *DDIT4L* in colorectal cancer.

## Discussion

RRBS is a method that combines genomic DNA digestion with size selection of target DNA fragments to examine genome-wide DNA methylation status, based on restriction enzyme enrichment of CpG-rich regions [10]. Previous studies assumed that DNA methylation of promoters [39,40] and CGIs [5,19] have the greatest functional significance in regulating gene expression; therefore, the current RRBS strategy retains *MspI* single enzyme digestion to reproducibly detect the methylation status of CGIs [16,41], although its coverage on other genomic elements is limited. However, DNA methylation in other genomic elements besides CGIs might also play important roles in gene regulation, as exemplified by recent studies on DNA methylation of CGI shores and introns. In one study, Irizarry et al. reported that 76% of tissue differential methylation regions (T-DMRs) among liver, spleen and brain, and most methylation alterations in colon cancer, occurred in CGI shores. Based on their results, the authors suggested DNA methylation of CGI shores might play a functional role in regulating alternative transcription during normal differentiation and cancer pathogenesis [22]. Furthermore, DMRs of introns also correlated with tumor aggressive behaviors by regulating alternative splicing [42] or the transcription of non-coding RNAs [25,43].

In the present study, the double-enzyme (*MspI* plus *ApeKI*) RRBS strategy reached almost full coverage of genomic elements of CGIs (~90%) and promoters (~80%), with more than 25 individual CpGs measurement, even if we only size-selected 40-220 bp fragments for sequencing. In particular, 13.0% of CGI shores and 10.6% of introns were additionally interrogated, reaching coverage of more than 70.1% of CGI shores and 40.1% of introns in comparison with the sRRBS (Figure 1a, 1b). Importantly, our results also indicated that the methylation levels of CGI shores, promoters or intron regions significantly varied with the coverage of individual CpG dinucleotides detected by the two RRBS strategies (Figure 2). In other words, the improved dRRBS identifies regional methylation levels more accurately because of its extensively increased coverage of genomic elements, and thus could be widely used as a new RRBS strategy in genome-wide identification of DNA methylation.

Despite the strategy of size-selection, the read length of high-throughput sequencing is an important factor influencing genomic coverage and data requirement for different RRBS strategies. Previously, we obtained about 3.4 M individual CpG dinucleotides using sRRBS with a PE50 sequencing strategy [21]. In the present study, we demonstrate that the dRRBS (*MspI* plus *ApeKI*) method is able to interrogate about 13.3% and 16.3% of the genome-wide CpGs using PE50 sequencing strategy with 40-220 bp and 40-300 bp inserts, respectively. As a comparison, it interrogates 16.6% and 21.4% of the genome-wide CpGs using a PE90 sequencing strategy with 40-220 bp and 40-300 bp inserts, respectively. Although longer read length could increase CpGs coverage, the majority of bases from the PE90 reads must be discarded as the reads were beyond the length ranges for many fragments. As a result, the required data for PE50 sequencing was about half that for PE90 sequencing to achieve the same depth. Thus, PE50 sequencing is a relatively efficient and cost-effective strategy for dRRBS. WGBS consumes micrograms of genomic DNA at a high cost; therefore, RRBS is more feasible for complex clinical samples. WGBS is a costly process, yet the costs of high-throughput sequencing are falling; therefore, nor only can we choose other enzyme combinations and size selection for dRRBS to obtain a much more comprehensive representation of the genome, but this method could also be applied to large scale studies, such as epigenome-wide association studies [44].

By applying this dRRBS method, we characterized the genome-scale DNA methylation at single-base resolution in colorectal carcinoma cell lines HCT116 and DKO. These cell lines have been employed as models by numerous researchers to validate the ability of their designed methods to detecting changes in DNA methylation [6,12], to study the expression of TSGs [32-36] or microRNA regulated by DNA methylation [24,37,45]. Interestingly, a recent study applied this cell model to identify genes that are prone to methylation in cancer cells. Taking advantages of genome-scale coverage and single-base resolution of our dRRBS technology, we extensively screened the DNA methylation status of these two cell lines. As expected, many CpG methylation changes were revealed from the comparison between HCT116 and DKO cell lines, which closely agreed with previous findings of HPLC-based global methylation analysis [27]. However, only a small amount of genes with significantly decreased methylation levels were significantly upregulated in DKO cells, which suggested that these genes might be repressed only by DNA methylation. These identified genes would be of interest for researchers in the study of DNMT-targeting mechanism and in the identification of TSGs. On the other hand, 16 genes were significantly hypermethylated in DKO cells, indicating that these loci might retain methylation due to a functional selection pressure, which is consistent with a recent study [28]. Taken together, profiling cytosine methylation in these two cell lines will provide basic data for researchers to study the DNA methylation status of thousands of genomic regions and to target those conserved methylation regions required for cancer cell survival using this cellular model.

## Conclusion

We have designed and validated systematically a double-enzyme RRBS strategy, which shows a considerable improvement over the previous single-enzyme RRBS method. This strategy can be applied to profile DNA methylation with a considerably increased whole-genome coverage and more accurate identification of methylation levels in various genomic regions. This method will significantly increase the ability of biomedical researchers to study genome-wide DNA methylation in multiple and complex clinical samples.

## Methods

### Sample preparation

A lymphoblastoid (YH) cell line was obtained from blood cells of the same individual whose genome and methylome (both by WGBS and RRBS) had been previously sequenced (YH) [21,31,46]. The genomic DNA of YH, a mature human dendritic cell (mDC) line, and the colorectal cancer cell lines HCT116 and DKO (DNMT1 −/−, DNMT3B−/−) generated from HCT116 cells with defective DNA methyltransferases *DNMT1* and *DNMT3b*, were isolated by TriReagent (Molecular Research Center, Cincinnati, OH), proteinase K digestion and phenol/chloroform extraction. Total RNA of HCT116 and DKO cell lines were extracted using TriReagent and treated with RNase-free DNase I (Promega) for 30 min, according to the manufacturer’s protocols. The integrity of total RNA was checked using an Agilent 2100 Bioanalyzer (Agilent Technologies).

### The single-enzyme RRBS library preparation

RRBS libraries with single *MspI* digestion were constructed for mDC cell line, as previously described [10,21]. Briefly, 100 ng of genomic DNA was digested with 300U of *MspI* enzymes (NEB) in 100 μl reactions at 37°C for 16–19 h. After purification, the digested products were blunt-ended, and then dA was added, followed by methylated-adapter ligation. To obtain DNA fractions of 40-120 bp and 120-220 bp ranges of *MspI*-digested products, two ranges of 160-240 bp and 240-340 bp adapter-ligated fractions were excised from a 2% agarose gel, respectively. Bisulfite conversion was conducted using a ZYMO EZ DNA Methylation-Gold Kit™ (ZYMO), following the manufacturer’s instructions. The final libraries were generated by PCR amplification using JumpStart^TM^ Taq DNA Polymerase (Sigma). RRBS libraries were analyzed by an Agilent 2100 Bioanalyzer (Agilent Technologies) and quantified by real time PCR.

### The double-enzyme RRBS library preparation

To construct the *MspI* and *ApeKI* digested RRBS library, 100 ng of input genomic DNA was assembled into 100 μl of reactions with 300 units of *MspI* (NEB) in 1× NEBuffer 2, incubated at 37°C for 7 h, and then 80°C for 20 min. Twenty units of *ApeKI* (NEB) were then added and incubated at 75°C for 16–20 h. After purification of the digested products with a MiniElute PCR Purification Kit (Qiagen), the following procedures were similar to that of *MspI* digested RRBS library construction. Briefly, to prepare samples for the ligation of 5-methylcytosine modified adapters, the overhangs generated by digestion were repaired in 100 μl reactions containing 15 units of T4 DNA polymerase, 2 units of Klenow fragment, 60 units of T4 polynucleotide Kinase, 0.6 mM of dNTP and 1× polynucleotide kinase buffer. Reactions were incubated at 20°C for 30 min, followed by purification. The blunt ended DNA fragments were then 3’ adenylated using 15 units of (3^′^ → 5^′^ exo-) (Enzymatics) and 0.2 mM of dATP in 50 μl reactions with 1× blue buffer at 37°C for 30 min. Purification with a MiniElute PCR Purification Kit (Qiagen) was performed and the products were resolved in 50 μl with a reaction mixture containing 360 units of T4 DNA ligase, 0.12 μM of 5-methylcytosine modified adapter oligo mix (5’ GATCGGAAGAGCACACGTCTGAACTCCAGTCAC and 5^′^ TACACTCTTTCCCTACACGACGCTCTTCCGATCT) and 1× Rapid ligation buffer. This was incubated at 20°C for 15 min, then 65°C for 15 min. After that, a 2% of agarose gel was used to separate the fractions of adapter-ligated products, and three ranges of 160-240 bp, 240-340 bp and 340-420 bp were excised and purified using a QIAquick gel extraction kit (Qiagen). Bisulfite conversion was conducted as described above and 200 ng of sheared unmethylated lambda DNA were used as carriers for each selected fraction in this treatment. Finally, the libraries were generated by PCR amplification in a reaction volume of 50 μl consisting of 10 μl of bisulfite converted products, 4 μl of 2.5 mM dNTP, 5 μl of 10× PCR buffer, 0.5 μl of JumpStart™ Taq DNA Polymerase, 2 μl of PCR primers and 28.5 μl of water. The following thermal cycling program was used: 94°C/1 min; 11 to 15 cycles of 94°C/10 s, 58°C/30 s and 72°C/30 s; and then extended for 5 min at 72°C and held at 12°C. PCR products were size-selected and purified using a QIAquick gel extraction kit (Qiagen). To assess the C-T conversion of bisulfite treatment, 50 pg of ummethylated lambda DNA was added into the genomic DNA samples together as input DNA. The libraries were analyzed by an Agilent 2100 Bioanalyzer (Agilent Technologies) and quantified by real time PCR.

### RRBS sequencing and data analysis

The libraries were sequenced using Illumina Hiseq2000 analyzer according to the manufacturer’s instructions. Raw sequencing data was processed by the Illumina base-calling pipeline. Low-quality reads that contained more than 30% ‘N’s or over 10% of the sequence with low quality value (quality value <20) per read were omitted from the data analysis. The clean reads were aligned to the UCSC human reference genome (hg18, ftp://hgdownload.cse.ucsc.edu/goldenPath/hg18/) in an unbiased way for bisulfite sequencing data: (1) all the observed cytosines were replaced by thymines and the guanines were replaced by adenosines *in silico*, forming two “alignment form” references; (2) observed cytosines on the forward read of each read pair were replaced by thymines, and observed guanines on the reverse read of each read pair were replaced by adenosines, *in silico*; (3) we then mapped the “alignment form” reads to the “alignment form” reference using SOAPaligner (http://soap.genomics.org.cn/). The uniquely aligned reads that contained at least one enzyme (*MspI* or *ApeKI*) digestion site at the ends were used and the first two bases (*MspI*) or first three bases (*ApekI*) on the 5^′^ end of the reverse reads that were filled in during the end-repair were masked for further analysis. Methylation levels of cytosines were analyzed as previously described [31]. DNA methylation alterations of promoters were analyzed statistically, as previously described [16]. Briefly, for each region, the number of methylated and unmethylated CpG reads was counted, a *chi*-square test was applied to identify differentially methylated genomic elements with a threshold of *p*-value < 0.01. Meanwhile, the difference in methylation levels between two samples should be more than 20%. CGIs were defined as regions greater than 200 bp with a GC fraction greater than 0.5 and an observed-to-expected ratio of CpG greater than 0.6. Promoters were defined as the regions spanning 2200 bp upstream and 500 bp downstream of the transcriptional start site. CGI shores were defined as regions of 2 kb in length adjacent to CGIs, and the enhancers were downloaded from VISTA Enhancer Browser (hg19, http://enhancer.lbl.gov/; the coordinates were transformed into hg18 version).

### DGE library construction and statistical analysis

For gene expression profiling of HCT116 and DKO cell lines, 4 μg of total RNA isolated from each sample was used DGE sequencing. The library was constructed as previously described [47], and subsequent statistical analyses were performed by the method of Audic and Claverie [48]. We collected a set of 11391 RefSeq genes for gene expression analysis (http://genome.ucsc.edu/, hg18), which can be detected by both the DGE and double-enzyme RRBS technologies. The significantly differentially expressed genes were determined at a threshold false discovery rate (FDR) <0.01 and two-fold reads per million (TPM) difference.

### Bisulfite genomic sequencing

PCR primers were designed by the online MethPrimer software (http://www.urogene.org/methprimer/index.html). The detailed information for their associated genes is listed in Additional file 1: Table S8. 400 ng of genomic DNA were converted using ZYMO EZ DNA Methylation-Gold Kit™ (ZYMO) and one-third of the elution products were used as templates. PCR amplification was carried out with a thermal cycling program of 94°C for 1 min; 30 cycles of 94°C for 10s, 58°C for 30s, and 72°C for 30s; and a final 5 min incubation at 72°C. PCR products were purified using the QIAquick Gel Extraction Kit (Qiagen) and subcloned. Twenty-four colonies for each PCR product were sequenced using the 3730 Genetic Analyzer (Applied Biosystems).

### Quantitative real time PCR

500 ng of total RNA was reverse transcribed using an oligo(dT)12 to 18 primer with Superscript II reverse transcriptase (Invitrogen), according to the manufacturer’s instructions. Reverse transcription PCR primers were designed between different exons to avoid any amplification of contaminating DNA (Additional file 1: Table S8) and the cDNA levels of target genes were analyzed using comparative Ct methods, and normalized to the level of β-actin. Real-time quantitative PCR reactions were performed on an ABI StepOne Plus Real Time PCR System (Applied Biosystems Inc.) using Eva Green (Biotium). The experiments were performed three times, independently, and the reactions were analyzed in triplicate.

## Abbreviations

RRBS: Reduced representation bisulfite sequencing; WGBS: Whole genome bisulfite sequencing; DMR: Differentially methylated regions; BSP: Bisulfite sequencing PCR; TSG: Tumor suppressor gene; CGI: CpG island.

## Competing interests

The authors declare that they have no competing interests.

## Authors’ contributions

JW led the experiments and YX led the bioinformatic analysis. LL prepared the HCT116 and DKO samples. YX and DG performed *in silico* analysis and analysis of data. HLL and NY participated in the data analysis. HW helped to design the analytical strategy. JW, YY and HJL performed RRBS, DGE, BSP and RT-PCR experiments. JW wrote the draft with editorial input from all authors. XZ contributed to the experimental design and to the data interpretation. FG and QT conceived and supervised the study and finalized the manuscript. All authors read and approved the final manuscript.

## Supplementary Material

Additional file 1 Contains all supplemental tables (Tables S1-8) and corresponding captions. Click here for file

Additional file 2 Contains all supplemental figures (Figures S1-7) and corresponding legends. Click here for file
