# Supplementary material for: Double restriction-enzyme digestion improves the coverage and accuracy of genome-wide CpG methylation profiling by reduced representation bisulfite sequencing
Source: BMC Genomics. 2013 Jan 16;14:11. doi: 10.1186/1471-2164-14-11 (PMC3570491; doi:10.1186/1471-2164-14-11)
Supplement: Additional file 2 — Contains all supplemental figures (Figures S1-7) and corresponding legends. [file 1471-2164-14-11-S2.pdf]

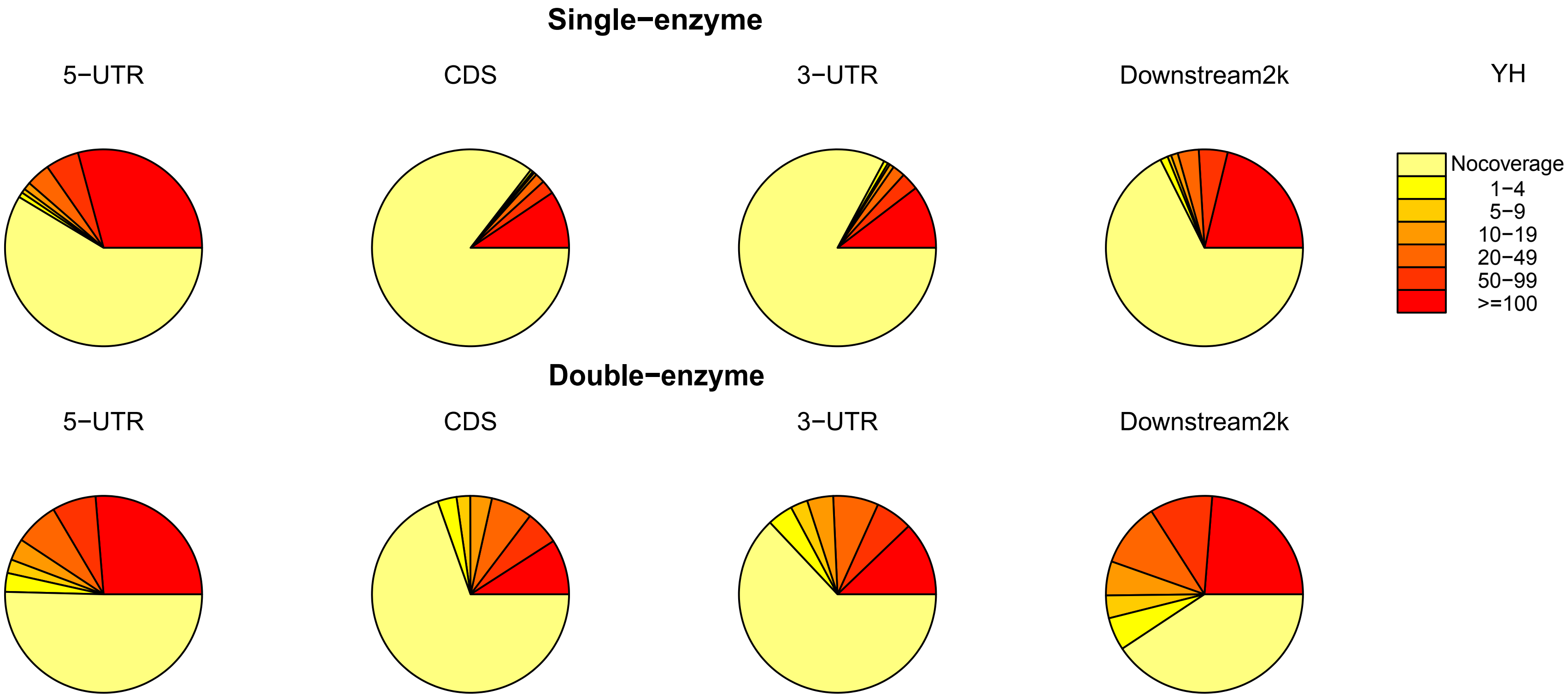

Figure S1 - Genomic coverage by single-enzyme (top) and double-enzyme (bottom) RRBS in the YH genome. With more than 25 individual CpGs measurements, the double-enzyme RRBS additionally detected about 1.2% of 5'UTR, 6.8% of CDS, 8.9% of 3'UTR and 13.5% of gene downstream 2kb regions.

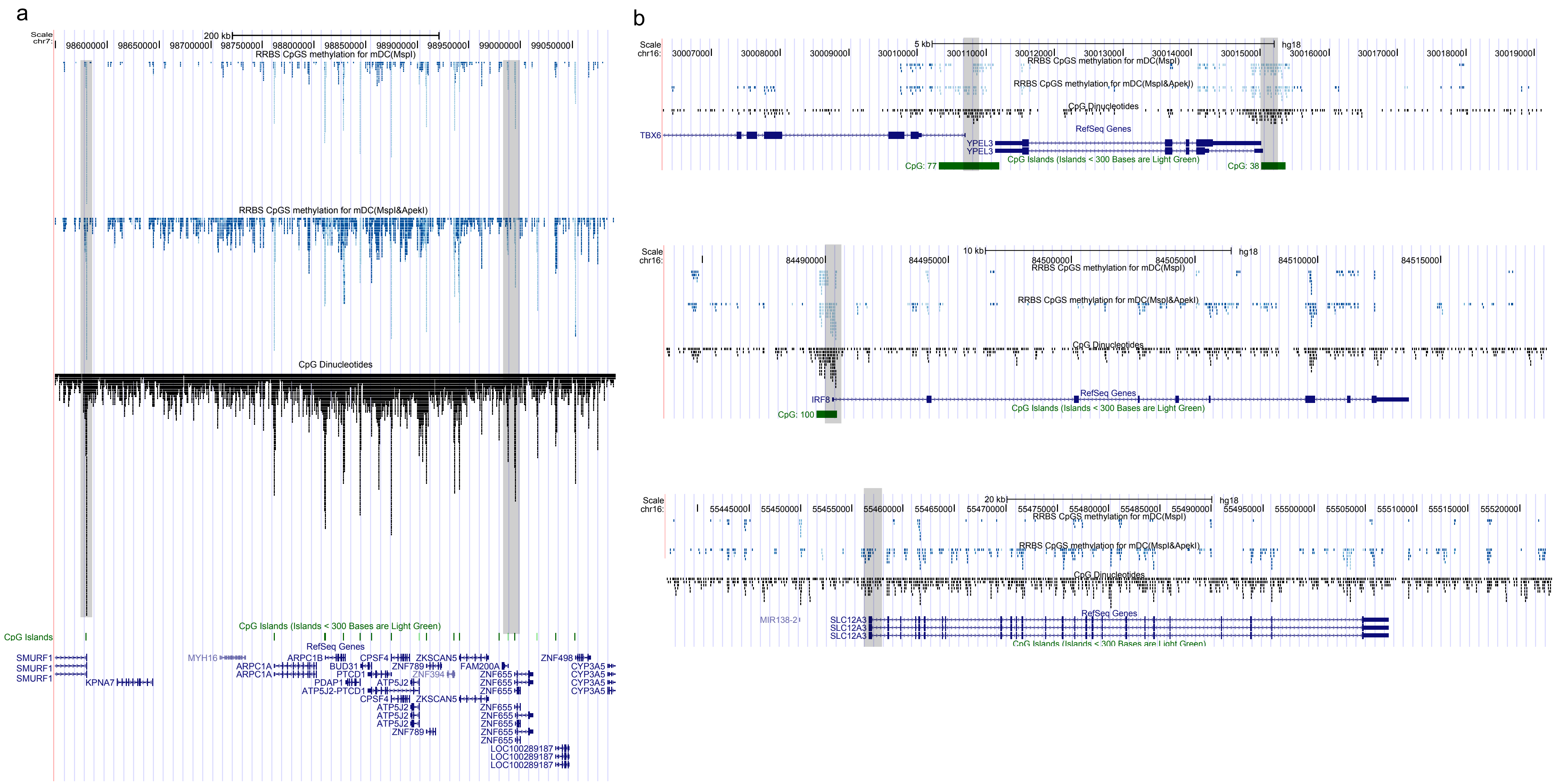

Figure S2 - Genomic CpG coverage by single- or double-enzyme RRBS based on in silico analysis or sequencing data in YH and mDC samples with all CpGs being covered 5 times (a) or 10 times (b). Ranges of size-selections for enzyme digested fragments and read lengths are displayed.

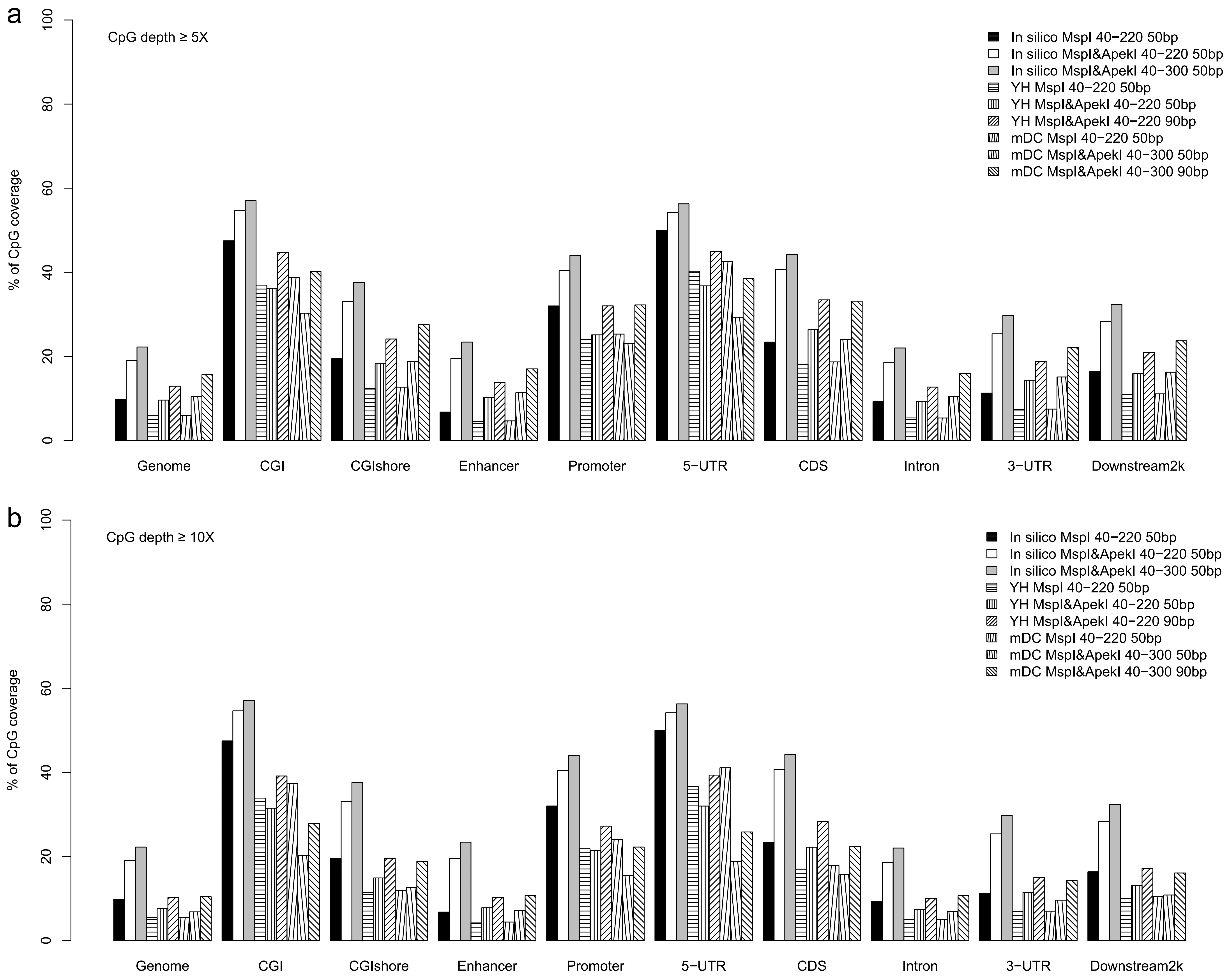

Figure S3 - DNA methylation in selected regions and genes. DNA methylation status of a randomly selected region, two TSGs with CGIs and one randomly selected gene without CGI from mDC cell lines detected by single- (MspI) and double-enzyme (MspI &ApekI) RRBS libraries are displayed by a UCSC Genome Browser screenshot. Each dot represents one CpG site, and the dark blue dots are more hypermethylated than light blue dots. The locations of CpG islands are represented with light green rectangles and the numbers of CpG contained in them are indicated.

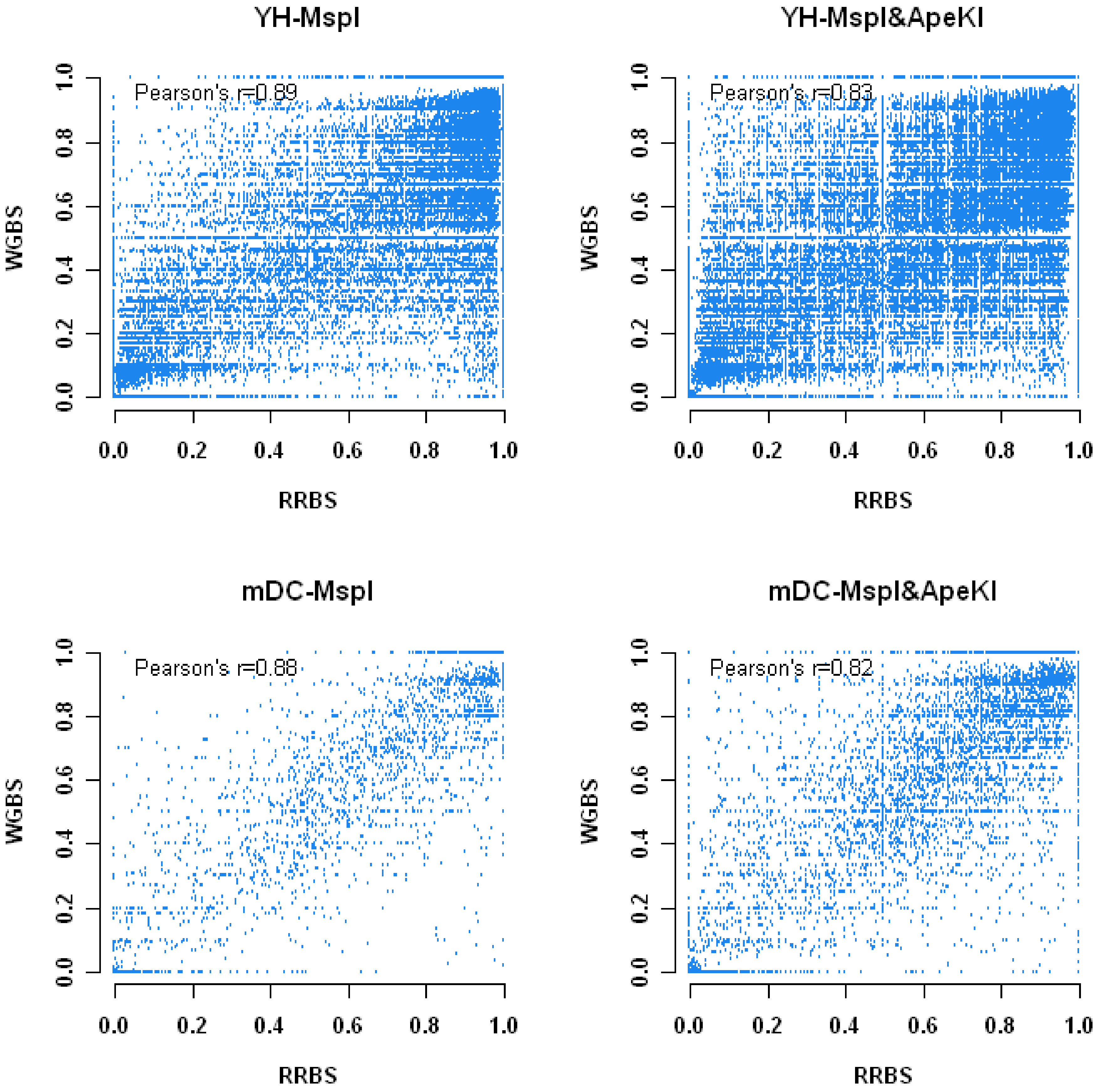

Figure S4 - Comparison of methylation levels for commonly detected CpG sites between the two strategies of RRBS and whole genome bisulfite sequencing (WGBS). Scatter plots between DNA methylation levels by WGBS and single- or double-enzymeRRBS in YH samples and mDC samples, respectively, are shown. The correlation coefficients were calculated based on Pearson correlation.

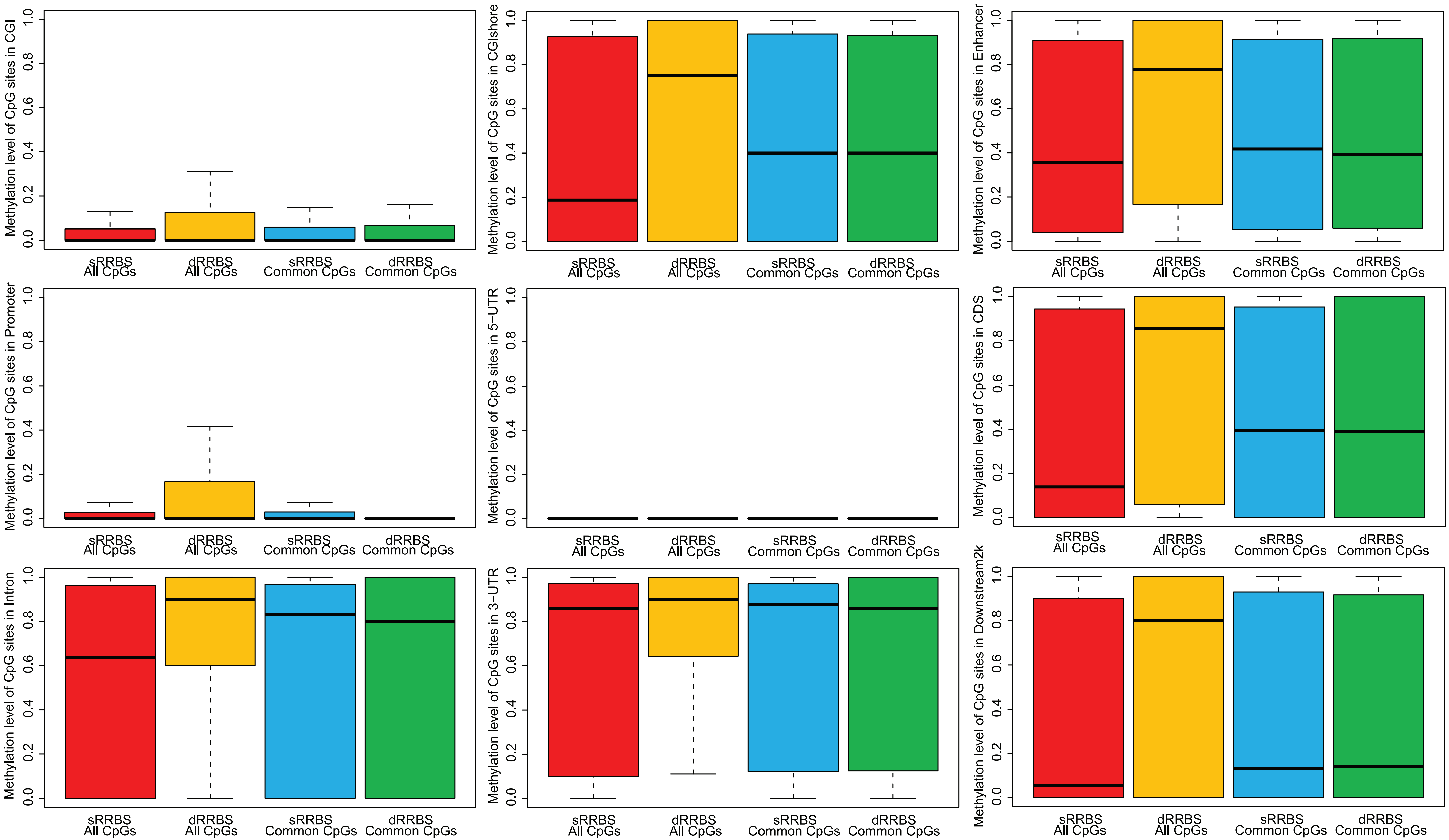

Figure S5 - Comparison of CpG methylation on CpG sites in each genomic element between single- and double-enzyme RRBS strategies.

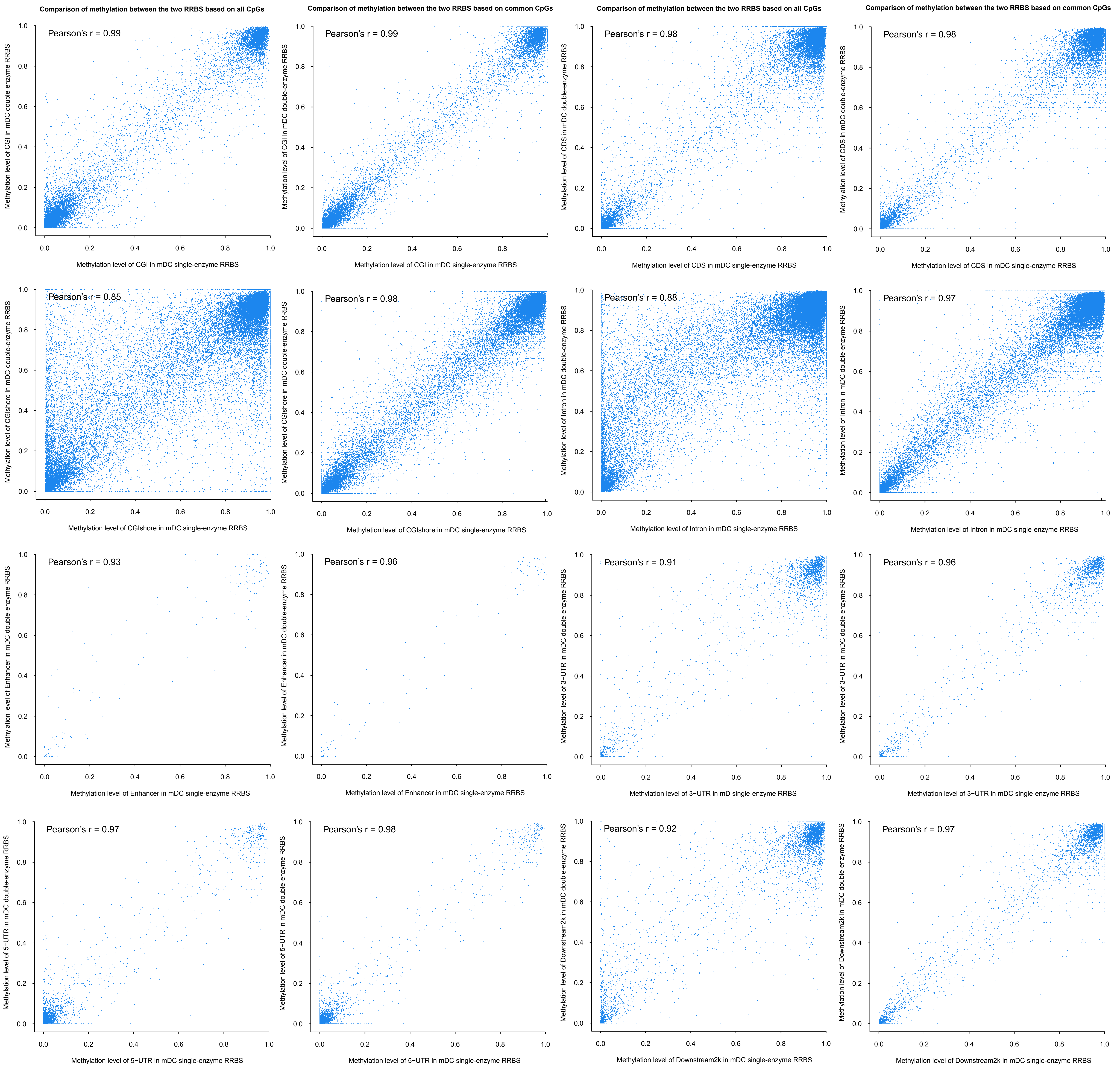

Figure S6 - Scatter plot for methylation levels of different genomic regions with all CpGs or with commonly covered CpGs detected by the single- or double-enzyme RRBS strategies. Each dot represents one genomic region.

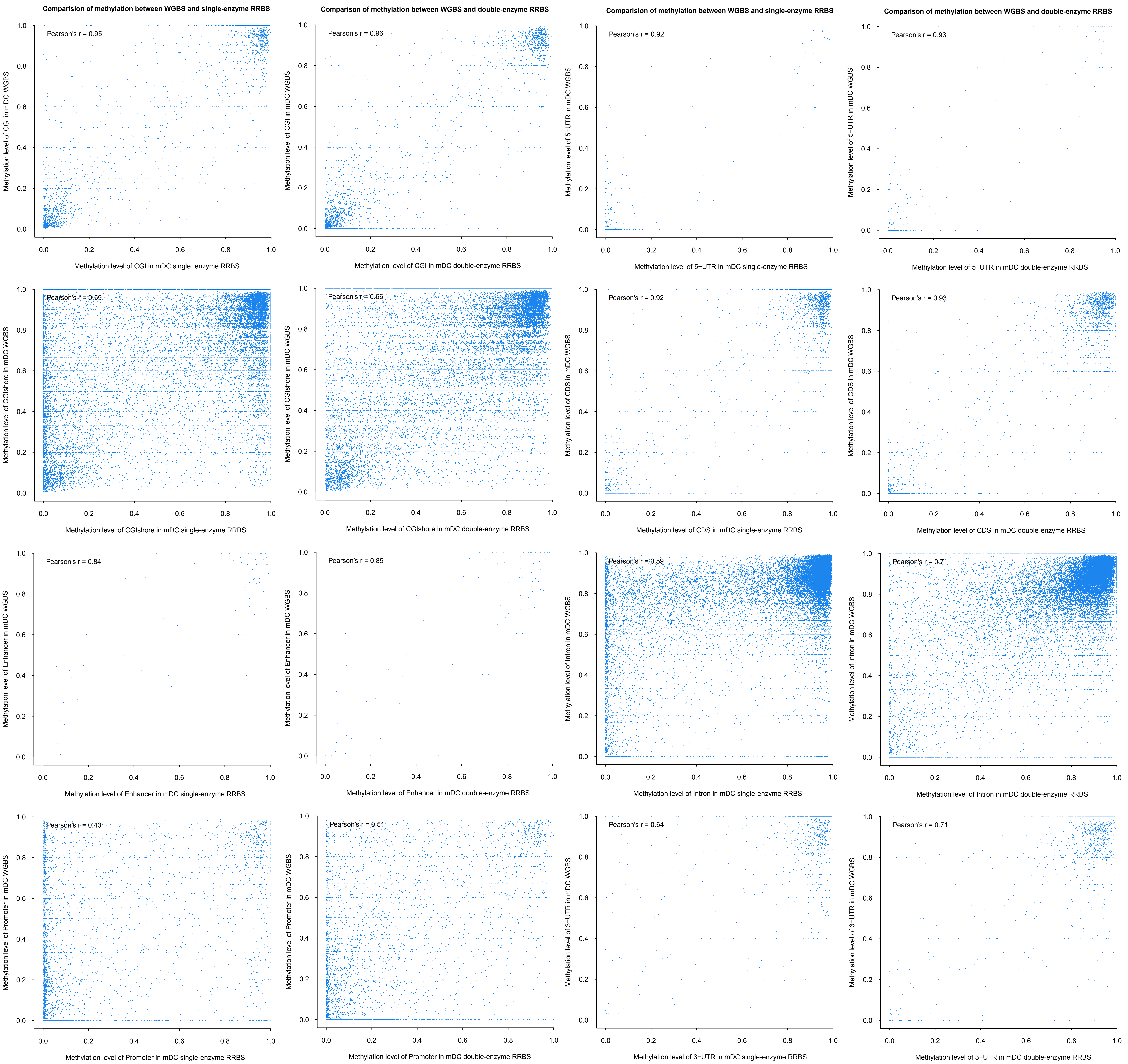

Figure S7 - Comparison of methylation levels of genomic regions by WGBS and by single- or double-enzyme RRBS, respectively. Higher Pearson correlation coefficients between double-enzyme RRBS and WGBS were observed.
